# Social dimensions of a forest-based bioeconomy: A summary and synthesis

**DOI:** 10.1007/s13280-020-01401-0

**Published:** 2020-10-13

**Authors:** Lea Ranacher, Ida Wallin, Lauri Valsta, Daniela Kleinschmit

**Affiliations:** 1 Wood K Plus - Competence Center for Wood Composites and Wood Chemistry, Kompetenzzentrum Holz GmbH, Altenberger Straße 69, 4040 Linz, Austria; 2 Chair of Forest and Environmental Policy, University Freiburg, Tennenbacherstr. 4, 79106 Freiburg i.Br., Germany; 3 Department of Forest Sciences, University of Helsinki, Latokartanonkaari 7, 00014 Helsinki, Finland

**Keywords:** Forest sector, Future directions, Political sciences, Stakeholder perceptions

## Abstract

How perceptions of the forest-based bioeconomy differ across country contexts and social groups is important as it opens possibilities for the development of more inclusive, locally and socially relevant bioeconomy policies and strategies. Therefore, this special section explores the social dimensions of the forest-based bioeconomy by focusing on discourses and perceptions of different actor groups in Europe. We introduce six articles that range from review and discursive approaches to consumer studies. The section adds to the existing literature by focusing not only on political decision makers, stakeholders, and experts but also on the public, media and students. Patterns in the presented discourses and perceptions can be identified but more is needed to validate these and respond to the question of representativeness.

## Introduction

Bioeconomy as a concept and political strategy conveys the basic idea of a sustainable economy where biobased and renewable materials have replaced non-renewables in all production stages. Forests are considered central suppliers of raw material in most European bioeconomy strategies. With high demands on the forest resource, conflicting perspectives on what forests should be used for are inevitable. Actors’ disparate perceptions and competing perspectives on the forest-based bioeconomy influence policies and ultimately impact future land use and forest management. Thus, empirical social science has an important role in uncovering dominant discourses and perspectives and creating possibilities for more comprehensive and critical perspectives on the forest-based bioeconomy.

This special section explores the social dimensions of the forest-based bioeconomy by focusing on discourses and perceptions of different actor groups in Europe. The articles included are grouped into two parts: Part 1 comprises articles on bioeconomy discourses and Part 2 covers empirical studies about perceptions. Below, we first situate our special section within the scientific literature on the bioeconomy topic and describe the socio-political context. We then briefly synthesize the main findings, introduce organizing questions and present conclusions about the social dimensions of the forest-based bioeconomy in Europe.

## The bioeconomy: A concept subjected to different perspectives and actor perceptions

Bioeconomy is an elusive concept that holds great promise for addressing some of the major global and national challenges of our times, such as climate change, biodiversity loss, and resource scarcity, whilst at the same time strengthening economic growth and national competitiveness (Pavone and Goven 2017). While political strategies worldwide present bioeconomy as a pathway towards a new and sustainable economy (Bioökonomierat 2015), serious concerns are increasingly being raised regarding the impact of bioeconomy on the environment. Whilst the bioeconomy is envisioned to generate economic and environmental benefits focusing on large-scale industrial processes, it is unresponsive to local sustainability goals (McCormick and Kautto 2013). The bioeconomy may result in a range of unintended ecological and social impacts resulting from the increasing use of biomass (Pfau et al. 2014). There is a growing body of literature (e.g. Mustalahti 2018) calling for monitoring of potential benefits and trade-offs associated with the increased use of biomass and for making the bioeconomy more socially inclusive. This is especially true for forests, which are considered central suppliers of biomass for the bioeconomy (Mustalahti 2018). Therefore, different conceptualizations of bioeconomy, and especially of the forest-based bioeconomy, have different potential impacts on land use and forest management.

In forest sectors worldwide, different and sometimes conflicting interpretations of the bioeconomy concept have emerged over the last decade (Kleinschmit et al. 2014). In fact, the meaning of bioeconomy seems to be in constant flux (Pülzl et al. 2014). The political vision of the bioeconomy enables new forms of cooperation as well as competition between established and new economic actors and also calls for new production and consumption patterns. Thus, the bioeconomy is subject to different actor networks (e.g. agriculture, forestry, energy biotech, healthcare, pharma) with a diversity of organizational strategies, interests, objectives and expectations, who are actively negotiating the future of the national bioeconomy. Which actors take part in shaping the bioeconomy discourse, and what views and arguments will ultimately prevail and thereby influence its trajectory is still unclear (Giurca 2020).

Still, national stakeholders’ perceptions and sectorial interests strongly influence the national interpretation of the bioeconomy concept and associated policies (Hodge et al. 2017). In particular, the national forest and wood-based sectors explicitly promise to be important suppliers of carbon neutral renewable materials and products for the bioeconomy (Swedish Forest Industries 2018). Representatives from the forest sector claim that their practices are carbon neutral and result in positive carbon uptake. These claims of sustainability have meant that negative environmental and social aspects of the forest-based bioeconomy have tended to be overlooked in national bioeconomy policies and strategies.

## State of the art, research gaps regarding social dimensions of the bioeconomy

Much forest-sector focused research has been conducted on the bioeconomy focusing on biotechnology innovations and production possibilities (Bugge et al. 2016). Societal and political dimensions have been identified as major research gaps in relation to the forest-based bioeconomy (Mustalahti 2018; Giurca 2019) and the bioeconomy in general (Stern et al. 2018). In particular, there is limited knowledge about societal inclusion, of how actors from the private sector, the general public, and consumers perceive the forest-based bioeconomy, and how these perceptions differ between countries and regions.

One recent study shows that while citizens and major brand owners are familiar with the concept of the bioeconomy, it hardly plays any role in the implementation, co-design or financing of bio-based products (Gerdes et al. 2018). As with other innovative products, bio-based products are facing the challenge of attracting consumers (McCormick and Kautto 2013). This is a potential drawback for producing and distributing bioeconomy goods and services. Understanding the perspectives of different actors is important for the development of bio-based products and their establishment on the market (Rogers 2003; Osterwalder and Pigneur 2010).

Social science research on the bioeconomy can improve the understanding of bioeconomy discourses and policies, their implementation, and the associated trade-offs between different perspectives. Thus, research focusing on societal priorities and communication in relation to the bioeconomy is needed (Gerdes et al. 2018). Broad acceptance of the bioeconomy is only possible if policies take into consideration social dimensions, conflicts, and debates (Meyer 2017). This particular perspective on how perceptions of the forest-based bioeconomy differ across country contexts and social groups is important as it opens possibilities for the development of more inclusive, locally and socially relevant bioeconomy policies and strategies.

Our special section intends to fill the knowledge gap described above and provides an overview of the diversity of social scientific approaches to discourses and perceptions on the forest bioeconomy. We start out from a broad understanding, defining the bioeconomy as *a form of economy that uses renewable, natural resources to produce food, feed, energy products and services* and the forest-based bioeconomy as *being an important sub-sector of the overall bioeconomy under which forests are projected to provide a significant contribution of biomass* (see e.g. McCormick and Kautto 2013; Kleinschmit et al. 2014).

This special section will provide readers with a spectrum of articles on (scientific and media) discourses and perceptions of forest bioeconomy across different European countries. These articles start out from both the political perspective (Wüstenhagen et al. 2007) and the marketing perspective (Pantano and Di Pietro 2012). In this kind of literature, the terms *awareness*, *perception*, *attitude* and *acceptance* are often used in different ways but generally refer to the meaning making of an issue and the acceptance or rejection of it (c.f. Assefa and Frostell 2007). Discourses and perceptions of the world around us are our mental impressions and understandings of the same and they ultimately guide our actions in the world.

Most importantly, this special section departs from the idea that the bioeconomy, and the role of forests in this transition, has no self-evident meaning. Bioeconomy as a term may refer to practices nurturing biodiversity, climate change adaptation in forest management, or local circular economies reflecting more bottom-up sustainability transformations. It is our aim to broaden and make visible the variety of perspectives of the forest-based bioeconomy in Europe.

## Short summary of articles and organizing structure of the special section

The articles included are grouped into two parts: Part 1 comprises articles on scientific and media discourses on bioeconomy and Part 2 covers empirical studies about perceptions amongst the general public and students. Before presenting the structuring questions of this special section, we first briefly describe these articles below.

### Part 1: Social dimensions of forest-based bioeconomy: Scientific and media discourse

Holmgren et al. (2020): This review article examines how social science literature focusing on forest-based bioeconomy transformations co-produces various imaginaries and pathways for reaching desired ends. Based on an interpretive analysis of 59 research articles, the authors conclude that much of the research tends to replicate a bioeconomy imaginary articulated in existing policies and strategies. In order to open up policy debates and offer alternative imaginaries of bioeconomy transformations, Holmgren et al. encourage scholars to adopt new strategies of inquiry.

D’Amato et al. (2020): Three main bioeconomy visions linked to ecosystem services are emerging in scientific literature: resource, biotechnology, and agroecology. This article encompasses a literature review where approximately half of the 45 documents reviewed are aligned with a resource vision of the bioeconomy, with emphasis on biomass production. Agroecology and biotechnology visions were less frequently found, but multiple visions tended to be present in each document.

Sanz Hernandez et al. (2020): Departing from the assumption that media play a key role in shaping public opinion, this article analyses the engagement of prominent Spanish stakeholders in forest bioeconomy debates in the media. The article provides new understandings of: (a) the degree of commitment of the different actors, (b) their spatial distribution, and (c) the way in which the media contribute to the creation of a collective framework for understanding and confronting the global challenges that the bioeconomy addresses.

### Part 2: Perceptions on the forest bioeconomy

Navrátilová et al. (2020): The potential for a fully developed forest-based bioeconomy remains untapped in Slovakia. Navrátilová and co-authors investigate public perceptions regarding the individual properties of renewable and non-renewable materials and how these perceptions relate to general bioeconomy development in the country. Natural renewable materials are strongly preferred among the respondents and this preference corresponds to support for the forest-based bioeconomy.

Masiero et al. (2020): This article presents an explorative survey of bioeconomy perceptions among 1400 students enrolled in 29 universities across nine European countries offering forestry programs. The analysis shows that around 70% of respondents have heard about the bioeconomy, mainly through university courses. Variation in the perceived importance of forestry for the bioeconomy is most pronounced across groups of countries along a North-South European axis. These results are relevant for developing pathways towards comprehensive bioeconomy curricula at European universities.

Kylkilahti et al. (2020): Consumer acceptance of Multi-Story Wooden Buildings (MSWB) is crucial for creating opportunities for businesses enacting this type of low-carbon urban housing. The authors explore Finnish students’ perceptions of MSWB and link these perceptions to ‘consumption styles’ through an online questionnaire. The results suggest that familiarity with the use of wood in housing is central to respondents’ perceptions. The familiarity of both environmentally minded and hedonic young consumers with the material contributes to a successful implementation of bioeconomy in the urban context.

## Framework and organizing questions of the special section

Reviewing existing social science literature on forest bioeconomy reveals some general findings that are used as an organizing structure to better understand discourses and perceptions of forest bioeconomy and to identify possible patterns. These results comprise notions about agents, sustainability and regional diversity. Thus, in the following sections, we briefly describe these findings and the resulting research questions that we address in this special section.

### Agents of change

The literature on bioeconomy has increasingly taken up the question of how agents are driving a transformation towards a bioeconomy. Studies have identified that although there are several actors involved in the bioeconomy and regional differences as well as changes over time, governments do have a central role within these networks (Giurca and Metz 2018; Korhonen et al. 2018; Giurca and Kleinschmit 2020). Some scholars have even described the bioeconomy as a “political project” (Goven and Pavone 2015). Governmental actors have a specific role in putting the concept of bioeconomy on the political agenda, initiating strategies and programs and selecting instruments for implementation. Private actors and academics as well as agents in the bioeconomy have been identified as being strongly interconnected with the government (Kröger and Raitio 2017; Giurca and Metz 2018; Korhonen et al. 2018). This may be due to the initial conceptualization of the bioeconomy as a knowledge-based bioeconomy where science was presented as the central (and main) component contributing to technical innovation. Other (societal) actors are marginalized in the discourse on bioeconomy, which has been problematized in several studies (e.g. Pülzl et al. 2014; Mustalahti 2018).

Whether political and governmental agency is also central in diverse discourses and perceptions is one question addressed in the contributions of this special section.

### Sustainability

Sustainability is addressed as one of the major goals of the bioeconomy in several political strategies and programs. It is highlighted that political bioeconomy strategies seek to achieve the Sustainable Development Goals with “green growth as a key goal” (Bioökonomierat 2018). Supporting development and economic growth through new and innovative products and at the same time having a positive impact on the environment is a prevailing frame of policy documents. This idea of a “win–win” situation whereby the bioeconomy simultaneously supports both the economy and ecology has been critically problematized by several social scientific studies (e.g. Kröger and Raitio 2017; Kleinschmit et al. 2017; Ramcilovic-Suominen and Pülzl 2018). Their results suggest that economy and technology are the central focus of political bioeconomy strategies while ecological and social aspects take a back seat (Pülzl et al. 2014).

How the sustainability of bioeconomy is addressed in the discourses and perceptions is the second question considered by the contributions of this special section.

### Disparities

So far, there have been few studies comparing bioeconomy strategies and discourses across different nations in Europe (Pülzl et al. 2014; Kleinschmit et al. 2017; Giurca and Kleinschmit 2020). Those that do exist conclude that the geographical focus influences the design of the forest bioeconomy concept. They indicate that the degree of importance placed on the (forest) based bioeconomy differs a great deal depending on the country—and how much political focus is devoted to the bioeconomy more generally. Furthermore, the framing of the bioeconomy, e.g. concerning dimensions of sustainability also differs between countries (Kleinschmit et al. 2017).

To understand whether there are certain patterns of geographical variations in the perceptions and discourses is the third question addressed by the contributions of this special section.

### Factors influencing perceptions of bioeconomy

There is a long-term acknowledgement within social science that there are several factors relevant for the construction of discourses and perceptions. Traditionally, socio-demographic factors, like age, level of education, gender and level of income have been validated as significant factors (e.g. Schell et al. 2012). However, depending on the discipline, factors selected in analysis can vary according to their aims. In this regard, sociological studies often differ from consumption studies.

Therefore, the last question addressed in the contributions of this special section relates to the diverse factors that have been demonstrated to have an impact on bioeconomy perceptions.

## Synthesis

The short summary of the contributions above shows that articles contributing to the special section cover a wide array of social dimensions of the forest-based bioeconomy. They range from review and discursive approaches to consumer studies. The reviews do not only provide the state of art on the social dimensions of bioeconomy but also present a snapshot of the international scientific discourse on the bioeconomy. A study on media outlines a different form of discourse and focuses on Spain. Two other contributions also have a specific country focus, Finland and Slovakia. Additionally, the article of Masiero et al. (2020) presents a cross-national analysis of Europe.

The studies of the special section do not present representative overarching results on the social dimensions of forest bioeconomy in Europe or on a specific country or group of actors. Instead, they provide an overview of the diversity of social scientific studies with a central focus on discourses and perceptions on the forest bioeconomy, thereby allowing for a more nuanced picture. With the organizing questions building the overarching framework of this special section, similarities and patterns across the studies are possible to uncover. Results will be presented in the following in line with the organizing questions.

### Political actors and governments as agents of change in the forest bioeconomy

Change agency in the forest bioeconomy can be observed in different ways. The most direct way is the perception of actors (be it self-perception or the perception of others) attributing agency. Another way is if the power of agents can be deconstructed, e.g. in form of ideas or institutionalization and resource allocation leading to mainstreaming or dominance in specific discourses and perceptions.

The former has been observed in the literature review of Holmgren et al. (2020), supporting findings of earlier studies in which political actors were perceived as central agents in the bioeconomy. Despite this finding, they also recognize that scientific articles have identified broad participation and inclusion as a prerequisite for a successful transformation. The central role of political and governmental actors is also mirrored in the article on the media discourse around the forest bioeconomy in Spain (Sanz-Hernandez et al. 2020). The authors conclude that the discourse is constructed by governmental actors with limited presence of other stakeholders, except for experts in media reporting.

Beyond this directly attributed agency, the articles also reveal that political ideas have been taken up. Holmgren et al. (2020) assume that social scientific literature follows the general ideas of a forest bioeconomy as presented in political strategies. Starting from an understanding that “social transformations are a long-term democratic project where definition of new socially shared meaning (…) and the inclusion of new actors are central (…)”, leads the authors to a critical conclusion that social scientific analyses of forest bioeconomy lack a broader and critical perspective.

The regional differences between the level of media attention paid to forest bioeconomy in Spain might also serve as an indicator of the power of political agency to stimulate discourses and perceptions. At the sub-national level, in some regions of Spain the bioeconomy is placed as a key issue on the political agenda. Correspondingly, media attention towards forest bioeconomy is higher in these regions than in others (Sanz-Hernandez et al. 2020). That the political attention and push for bioeconomy has an indirect effect on awareness and perceptions is also supported by the study of Masiero et al. (2020). Results from the cross-national comparison of students’ perceptions show, not surprisingly, that those countries that have been early political starters with bioeconomy strategies in Europe (in particular Finland) have advanced university programs addressing bioeconomy further than others. This is in turn corresponds with greater knowledge about bioeconomy and a higher level of satisfaction with these programs amongst students.

### Sustainability

The issue of sustainability is central in the different articles of this special section. However, there are significant differences in the approaches taken. Some articles start from the assumption that replacing fossil-based materials and products is in general a first step in transformation towards sustainability that serves ecological needs (Navrátilová et al. 2020; Kylkilahti et al. 2020). Holmgren et al. (2020) confirm that this normative starting point, whereby sustainability is limited to the use of renewable bio-based products replacing fossil-based products and hinting at long-term sustainable yield of forest biomass, is used in the social scientific discourse on bioeconomy. Others start from a more open, partly critical perspective (D’Amato et al. 2020; Sanz-Hernandez et al. 2020) assessing whether and how sustainability is addressed in certain fora or by specific groups.

The former approach is supported by the general trend of transformation towards bioeconomy as a response to overcome negative impacts of non-renewable resources on the environment. Navrátilová et al. (2020) acknowledge that consumers in Slovakia see the need to minimise negative impacts of materials and products on the environment and thus the imperative of sustainability. They therefore seek a move to renewables. This starting point is accompanied by the increasing woody biomass consumption in Slovakia, which the authors predict will become the most important renewable energy source in the future. Their assumption is mirrored by their survey results, showing that the public attributes eco-friendliness to wood material while considering that non-renewables are not eco-friendly. Surprisingly, and contradictory, other bio-based materials are perceived as not being as eco-friendly as wood. The authors assume that a lack of information might be the reason for this difference. The article of Kylkilahti et al. (2020) follows a similar direction and comes to a similar conclusion. Their survey shows that multi-storey wood buildings (MSWB) are generally perceived in Finland to be environmentally friendly. However, this attribute does not stand alone and seems to be strongly connected to the aesthetics of MSWBs. The case of wood materials in Slovakia being viewed as more eco-friendly than other bio-based materials might similarly be affected by other attributes of this resource.

The other articles of this special section approach sustainability more openly. They reconfirm results of earlier studies presenting the bioeconomy discourse and perceptions as focusing in particular on economic growth rather than on the full spectrum of sustainability. For example, the article of Sanz-Hernandez et al. (2020) shows that the Spanish media discourse describes forest bioeconomy as an opportunity for economic growth while at the same time allowing for increased resilience to climate change, prevention of forest fires and promotion of regional development. This way of framing the bioeconomy presented in policy programs and strategies in diverse EU member states is described as a win–win solution, with economic growth and development being in line with environmental protection and attempts to respond to negative environmental impacts (Kleinschmit et al. 2017). Critical or negative reporting about bioeconomy are neglected in the Spanish media. This seems to indicate that the narrative provided by political strategies has been mainstreamed in the media discourse.

The review of literature connecting bioeconomy and ecosystem services analysed by D’Amato et al. (2020) confirms results of earlier studies that issues like biomass production, biotechnology and agroecology are particularly central in the scientific discourse. Both technological and economic issues are more often addressed than all others within the reviewed articles. In contrast, the more general question of the sustainability of bio-based processes, production and services is addressed only to a minor degree. Some of the reviewed articles additionally express scepticism about sustainability assessments as an appropriate instrument to evaluate sustainability.

Results from the cross-country student survey mirror this framing of bioeconomy, showing that bioeconomy lectures in universities are mainly embedded in economic and technology courses as opposed to social science courses (Masiero et al. 2020). Therefore, it might not come as a surprise that forest students perceive bioeconomy first and foremost as an opportunity for the forest sector, with students in only Germany and Austria also raising some concerns about sustainability (ibidem).

### Divergence in the use of the bioeconomy concept

This special section reconfirms the divergence in the use of the bioeconomy concept that has been identified earlier in the literature. However, different patterns within this divergence can be identified. On the one hand, differences in the use of the concept are based on the different design or framing of the bioeconomy, which for the case of Europe has already been identified in diverse studies (Kleinschmit et al. 2017; Ramcilovic-Suominen and Pülzl 2018). On the other hand, regional disparity relates to the fact that the level of attention paid to the (forest) bioeconomy differs depending on regions, countries, the natural resources available and other factors.

A high level of geographical spread internationally is an indicator of (political) attention paid to the bioeconomy, with about 50 countries having already established a bioeconomy strategy, including countries in South America and Asia (Bioökonomierat 2018). In contrast, the focus of social scientific studies on bioeconomy is less widespread but rather dominated by articles from authors with affiliations in northern industrial countries, particularly from Northern Europe (Finland, Sweden and Germany) (Holmgren et al. 2020). D’Amato et al. (2020) highlight that this is not only true for purely social scientific studies but also for those dealing with the interlinkage between bioeconomy and ecosystem services. This article shows that the authors of the reviewed articles are from the same countries, the US, Germany and Finland, with co-authorship strongly interconnected within a European network.

The article of Masiero et al. (2020) adds another dimension to these results by zooming into the perceptions of forest students. As students in the field of natural resources, they not only represent potential future stakeholders of the forest bioeconomy but it can also be assumed that they are more exposed to information and knowledge about natural resources and to the bioeconomy than other groups in the population. Surprisingly, the survey results show that still one third of the responding students have not heard about the bioeconomy. Masiero et al. (2020) have uncovered that amongst the students, the awareness of the bioeconomy concept differs along a north-south gradient in Europe, with highest awareness of and knowledge about the bioeconomy amongst students in the Northern European countries (particularly Finland) and only low awareness in the Southern European countries. Correspondingly, the level of satisfaction with the university programs on bioeconomy is far higher in Finland than in Spain and France. The scarce penetration of forest bioeconomy in the media in Spain recognized by Sanz-Hernandez et al. (2020) adds to this picture. It can be assumed that the attention paid to bioeconomy in the media as well as in universities (and correspondingly by students) in Spain is not as high as in other European countries where bioeconomy has already been stressed and pushed for a long time.

Apart from the national setting of bioeconomy, disparities between perceptions can also result from the place of residence. Navrátilová et al. (2020) identified that rural residents have a much more positive perception of wood and other natural materials than the urban population. Hence, not only might the political focus on bioeconomy cause asymmetries in awareness and perceptions but also peoples socio-cultural and geographic situation.

### Factors relevant to perception

This special section has provided a broad overview of studies investigating the social dimension of the bioeconomy that start from different disciplinary backgrounds and assumptions. According to these, the authors have focused on different factors that describe and explain the construction of the discourses and perceptions at hand. Geographical divergence as one major factor explaining variations in awareness and perceptions of bioeconomy is already introduced above.

Other socio-demographic factors relevant for explaining differences in the perception addressed in the contributions to this special section are gender, age, pre-existing level of knowledge, previous exposure and consumption style. They generally reconfirm already existing results. For example, assessing the factor gender in their article, Navrátilová et al. (2020) show that women are more focussed on environmental factors. Similarly, the same study validates the pattern that with increasing age people tend to show higher preference for wood materials than for other products.

The article of Kylkilahti et al. (2020) adds that it is not only consumers’ ecological attitudes that play a role in their perception of MSWB but that their perceptions also differ in relation to other factors, e.g. knowledge and childhood experience of MSWB. In the case of the latter, a more positive attitude amongst those who have been exposed to the product earlier in life is observable.

## Conclusion

The special section on the social dimension of bioeconomy provides a broad overview of studies in these areas with particular focus on discourses and perceptions amongst different groups. It adds to the existing literature by not only focusing on political decision makers and stakeholder/experts but also on the public, media and (forest) students. This not only adds a different perspective but is also highly relevant as earlier studies have demonstrated that there are significant differences between the perceptions of the public and experts, particularly in relation to forest and forest products (Lim et al. 2015) and forest-based sector activities (Ranacher et al. 2017).

However, these “non-experts” are not independent from the discourses of other actors, and especially from political settings. According to results of earlier studies, the contributions of this special section suggest that political attention towards bioeconomy has an effect on awareness and perception amongst other groups. This might also contribute to the dominant framing of sustainability in bioeconomy discourses and perceptions that highlights economic and technological dimensions before others. The “win-win” framing presented by the contributing articles corresponds with those of political strategies and programmes. The geographical disparity can also be interpreted as being linked to the political awareness. In countries or regions that have for several years been forerunners in stressing a transformation towards bioeconomy, this seems to affect the awareness in other social areas, as in the example of universities and students. Besides the assumed significant impact of political push towards the bioeconomy, other factors – such as gender, age, knowledge or former exposure—also have a significant impact on perceptions.

All in all, this special section shows that despite the diversity of contributions, patterns in the presented discourses and perceptions can be identified. However, to confirm these, research on these specific patterns is needed to validate these results and respond to the question of representativeness.
